# Astragalus polysaccharide‐containing 3D‐printed scaffold for traumatized skin repair and proteomic study

**DOI:** 10.1111/jcmm.70023

**Published:** 2024-08-19

**Authors:** Weibin Du, Zhenwei Wang, Meichun Han, Yang Zheng, Bowen Tao, Ningfang Pan, Guanai Bao, Wei Zhuang, Renfu Quan

**Affiliations:** ^1^ Research Institute of Orthopedics The Jiangnan Hospital affiliated to Zhejiang Chinese Medical University Hangzhou Zhejiang China; ^2^ Hangzhou Xiaoshan Hospital of Traditional Chinese Medicine Hangzhou Zhejiang China; ^3^ Health Science Center, Ningbo University Ningbo Zhejiang China; ^4^ Pain and Rehabilitation Medicine Zhejiang Cancer Hospital Hangzhou Zhejiang China

**Keywords:** 3D‐printed scaffold, Astragalus polysaccharide, proteomic study, traumatized skin repair, YAP/TAZ signalling pathway

## Abstract

Astragalus polysaccharide‐containing 3D‐printed scaffold shows great potential in traumatic skin repair. This study aimed to investigate its repairing effect and to combine it with proteomic technology to deeply resolve the related protein expression changes. Thirty SD rats were divided randomly into three groups (*n* = 10 per group): the sham‐operated group, the model group and the scaffold group. Subsequently, we conducted a comparative analysis on trauma blood perfusion, trauma healing rate, histological changes, the expression of the YAP/TAZ signalling pathway and angiogenesis‐related factors. Additionally, neonatal skin tissues were collected for proteomic analysis. The blood perfusion volume and wound healing recovery in the scaffold group were better than that in the model group (*p* < 0.05). The protein expression of STAT3, YAP, TAZ and expression of vascular‐related factor A (VEGFA) in the scaffold group was higher than that in the model group (*p* < 0.05). Proteomic analysis showed that there were 207 differential proteins common to the three groups. Mitochondrial function, immune response, redox response, extracellular gap and ATP metabolic process were the main groups of differential protein changes. Oxidative phosphorylation, metabolic pathway, carbon metabolism, calcium signalling pathway, etc. were the main differential metabolic pathway change groups. Astragalus polysaccharide‐containing 3D‐printed scaffold had certain reversals of protein disorder. The Astragalus polysaccharide‐containing 3D‐printed scaffold may promote the VEGFs by activating the YAP/TAZ signalling pathway with the help of STAT3 into the nucleus, accelerating early angiogenesis of the trauma, correcting the protein disorder of the trauma and ultimately realizing the repair of the wound.

## 1 INTRODUCTION

The skin is the largest organ in the human body and it serves several functions, the most important of which is to act as a barrier separating the internal organs of the body from the external environment.^1^, ^2^ The physiological process of wound healing occurs immediately once the protective barrier formed by the epidermis and dermis is broken.^3^ Healing of skin injuries requires a lengthy process. If not intervened promptly, it may lead to insufficient local blood supply and subsequent infiltration of inflammatory cells, which induces muscle contraction, severely affecting wound healing and may even evolve into chronic wounds. How to accelerate wound healing has become a therapeutic challenge of universal concern worldwide.^4^ The elasticity, mechanical heterogeneity and topography of the extracellular matrix (ECM) play a crucial role in the speed and quality of wound healing. The ECM is mainly composed of proteins, which often require extensive post‐translational modifications to function.^5^, ^6^, ^7^, ^8^ Comprehensive coverage and quantitative assessment of the skin can be achieved through proteomic methods, and mass spectrometry‐based proteomic studies will greatly advance our understanding of skin pathophysiology and help elucidate molecular disease mechanisms.

Tissue‐engineered skin substitutes have been widely used to promote skin wound healing,^9^ and various types of tissue‐engineered dermal substitutes have been used in clinical practice.^10^ Compared with conventional treatments, artificial dermis can be more effective in promoting cell growth towards dermal tissue. However, tissue‐engineered dermal substitutes continue to face numerous limitations, such as low survival rates post‐transplantation, susceptibility to immune rejection, lack of skin elasticity, loss of appendages and sensory deficits. These factors significantly impact the functionality and quality of life of patients following wound healing.^11^, ^12^ 3D bioprinting is an emerging biomaterials fabrication technology, which can precisely distribute biological materials, active cells and other components, and build complex spatial structures.^13^ With the maturity of high‐precision 3D bioprinting technology, various types of hydrogel materials have been used for skin 3D bioprinting.^14^ Collagen has low immunogenicity and can provide a good microenvironment, which is more conducive to cell proliferation and targeted differentiation.^15^ Sodium alginate is beneficial to maintain a moist wound environment can absorb wound exudates and also increase the formability of the scaffold.^16^, ^17^ Silk fibroin protein has good biocompatibility and mechanical properties, which can enhance the elasticity and spatial structure of the scaffold and better cooperate with the growth of cells.^18^ Meanwhile, more and more studies have demonstrated the important role of angiogenesis in skin repair.^19^, ^20^, ^21^ Astragalus polysaccharide is one of the main active substances in Astragalus, which has great potential in anti‐inflammatory, vascular protection and angiogenesis promotion. Zhang G et al. have found through experimental studies that Astragalus polysaccharide can significantly enhance the migration of vascular endothelial cells and the formation of the lumen, suggesting that Astragalus polysaccharide can promote tissue repair by promoting vascular regeneration.^22^, ^23^

Therefore, we prepared Astragalus polysaccharide‐containing 3D‐printed scaffold and transplanted them into whole skin defect wounds in rats to explore their reparative effects, and combined them with proteomics technology to deeply resolve the relevant protein expression changes and provide a new strategy for the treatment of skin trauma.

## 2 METHODS

### 2.1 Experimental animals

Thirty male SD rats, weighing 160 ± 20 g, were grouped and acclimatized at the animal experimentation centre of Zhejiang Chinese Medical University for 1 week for the experiments. Purchase and feeding, and other animal procedures followed the animal research guidelines of the National Institute of Health and the Animal Research Committee. It was approved by the experimental animal ethics committee of the Zhejiang Chinese Medical University (No. IACUC‐20220627‐27).

### 2.2 Model preparation

Based on the previous modelling foundation of the research group, the whole skin defect model was prepared. After anaesthesia, the modelling area (2 cm on both sides of the spine) was shaved and depilated. After disinfection with iodine, a 1 × 1 cm square model of the whole skin defect was prepared with surgical scissors. Postoperatively, the wound was hemostatised and kept dry to prevent wound infection.

### 2.3 Grouping and treatment

Thirty SD rats were randomly divided into three groups of 10 rats each according to the random number table method, as follows: sham operation group, model group and scaffold group. (1) Sham‐operated group: the modelling area was only clipped and no trauma model was prepared. (2) Model group: prepare a model of full‐layer skin defect, disinfect the wound with iodine povidone and bandage it with gauze to prevent wound infection. (3) Scaffold group: transplant Astragalus polysaccharide‐ collagen‐sodium alginate‐silk fibroin 3D‐printed scaffold on the same day (the scaffold preparation protocol used our group's pre‐mature technology.^24^ Briefly, sodium alginate and silk fibroin were mixed in proportion and then 3D printing parameters were set, and the scaffold was prepared by a 3D printer. Finally, 0.2% type I collagen and 200 μg/mL of Astragalus polysaccharide solution were dropped on the scaffold.), gauze dressing to prevent wound infection.

### 2.4 Scanning electron microscopy detection

Astragalus polysaccharide‐containing 3D‐printed scaffolds were fixed in 2.5% glutaraldehyde solution at 4°C overnight. After the steps of fixation in 1% osmic acid solution, dehydration of the samples in ethanol solution with gradient concentrations (including 30%, 50%, 70%, 80%,90% and 100%), and the treatment of the samples with a mixture of ethanol and isoamyl acetate. After critical point drying and platinum spraying, the internal structure of the scaffold was observed under a scanning electron microscope.

### 2.5 Cell viability staining assay

Fibroblasts were added to each 3D printed scaffold according to a suspension of 1 × 106cells/ml of 100ul. After 5 days of culture, the cell growth was observed under the microscope and Calcein‐AM/PI double staining was selected, after 30 min of staining, the live cells (green fluorescence) and dead cells (red fluorescence) were detected under the fluorescence microscope using 490 nm wavelength excitation filters.

### 2.6 Laser Doppler perfusion imaging detection

Laser Doppler perfusion imaging was used in each group at 0, 7 and 14 days, with a distance of 10 cm between the probe and the test object and an imaging range of 1.0 × 1.0 cm, and PIMSoft software was applied to record and analyse the body surface blood flow maps. Compare the changes in blood perfusion in each group.

### 2.7 Postoperative wound observation

The wounds of the model group and the scaffold group were photographed at 7 and 14 days, and the wound healing was analysed using image‐pro Plus 6.0 image analysis software. Wound healing percentage = [(original wound area − wound area at the time of observation)/original wound area] × 100%.

### 2.8 Histological testing

The neonatal skin tissues in the modelling area were taken from each group after 7 days, fixed with 4% PFA solution for more than 24 h, dehydrated, paraffin‐embedded and made into 4 μm sections. HE and Masson staining were performed and the sections were dehydrated and sealed after completing the steps, observed under the microscope and photographed for comparison.

### 2.9 Immunofluorescence detection

After 7 days in each group, neonatal skin tissues were taken from the modelling area and frozen sections were made. 4% PFA solution was used for fixation, endogenous peroxidase was removed, 5% BSA was used for closure and the cells were incubated with primary antibody VEGFA (dilution ratio 1:200) and CD31 (dilution ratio 1:200) at 4°C overnight, PBS was washed three times, then the secondary antibody was incubated at room temperature, and the nuclei of the cells were restained with DAPI, and the final rinsed and sealed slices of the slices were rinsed and sealed with PBS, and then observed and photographed under the fluorescence microscope.

### 2.10 Western blot detection

The neonatal skin tissues in the modelling area were taken from each group after 7 days, and the expression of target proteins in each group was compared after the steps of extracting the total tissue proteins, protein concentration measured by BCA method, SDS‐PAGE electrophoresis, membrane transfer, incubation with primary antibodies (STAT3, YAP, TAZ and VEGFA, dilution ratio 1:1000) and secondary antibodies, chemiluminescence, photographs taken on the machine and software analysis.

### 2.11 Protein sample preparation and processing

Neonatal skin tissues were taken from the modelling area after 7 days in each group. Cut, add the appropriate amount of protein lysis buffer and use a high‐throughput tissue grinder to shake three times, each time for 40s. Lysis was performed on ice for 30 min, during which it was mixed every 5 min for 5–10s. Centrifugation was performed at 12000*g* for 30 min, and the supernatant was collected. Protein content was determined by the BCA method. Preparation of protein sample, 100 μg of protein sample was taken, lysis buffer was replenished and proteolysis was performed according to the procedure. Peptide desalting was performed with HLB and peptide quantification was performed using Thermo Fisher Scientific Peptide Quantification Kit.

### 2.12 4D‐DIA mass spectrometric detection

Aliquots of peptides were dissolved using mass spectrometry up‐sampling buffer and analysed by LC–MS/MS. Mobile phase A: 2% acetonitrile 0.1% formic acid, mobile phase B: 80% acetonitrile 0.1% formic acid. Separation gradient: 0–70 min, mobile phase B increased from linear 5 to 23%; 70–90 min, mobile phase B increased from 23% to 29%; 90–100 min, mobile phase B increased from 29% to 38%; 100–102 min, mobile phase B increased from 38% to 48%; 102–103 min, mobile phase B increased from 48% to 100%; 103–120 min, mobile phase B linearity was maintained at 100%. Mass spectrometry analysis was performed using *Q*‐Exactive HF‐X.

### 2.13 4D‐DIA data analysis

The 4D‐DIA raw data were imported into the Spectronaut™ software system for library search analysis. iRT correction retention time, and six peptides per protein and three sub‐ions per peptide were selected for quantitative analysis. The parameters were as follows: Protein FDR ≤0.01, Peptide FDR ≤0.01, Peptide Confidence ≥99%, XIC width ≤ 75 ppm, excluding shared peptides and modified peptides and calculating the peak area sum to obtain the quantitative results.

### 2.14 Bioinformatics analysis

The *t*‐test function in R language was used to calculate the significance *p*‐value and fold change (FC) of differences between groups. The significance test *p* < 0.05, and also the proteins with a multiplicity of difference greater than 1.2 times were differentially expressed proteins. The Gene Ontology database (http://geneontology.org/) was selected to perform GO annotation analysis functional clustering analysis for all differential proteins in terms of biological processes, cellular components and molecular functions; the KEGG (http://www.genome.jp/kegg//) pathway database was used to perform the metabolic pathways involved in the differential protein analysis.

### 2.15 Statistical analysis

All of the experimental results were expressed as the mean ± SD (standard deviation). All statistical analyses were performed using SPSS 21.0 software. The significance of differences between groups was determined by a two‐tailed unpaired Student's *t*‐test or one‐way ANOVA with Dunnett's post hoc test when samples were not distributed normally. A value of *p* < 0.05 was considered to be statistically significant.

## 3 RESULTS

### 3.1 Results of scanning electron microscopy and cell viability staining

**FIGURE 1:**
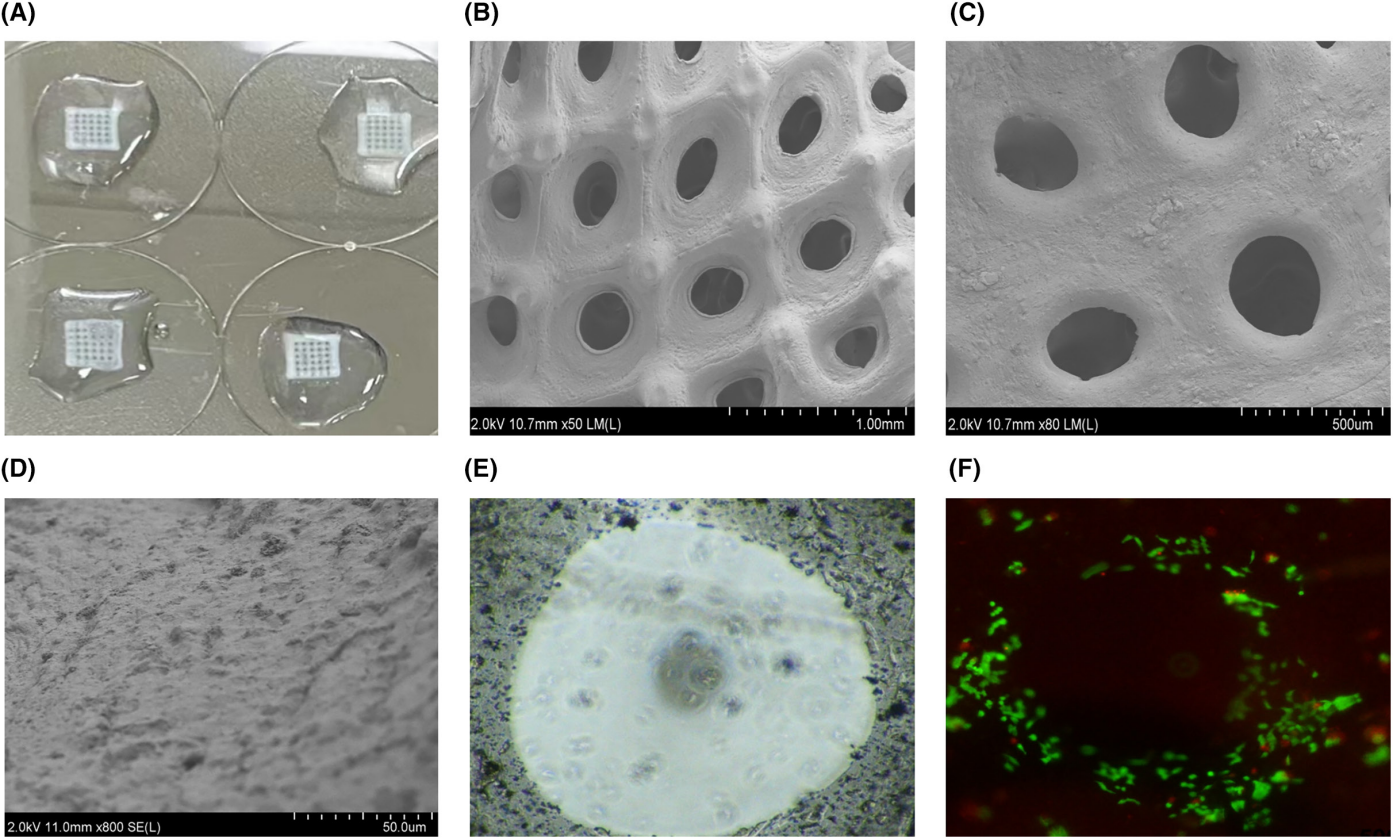
(A) General view of the 3D printed scaffold containing Astragalus polysaccharide. (B–D) The 3D‐printed scaffold containing Astragalus polysaccharide was observed under a scanning electron microscope. (E) The co‐culture of fibroblasts and 3D‐printed scaffolds containing Astragalus polysaccharide was observed under the microscope. (F) The result of Calcein‐AM/PI Double Stain.

The scaffold was in the form of a white transparent grid, the scaffold apertures were of uniform size and spacing, the thickness was uniform and the surface of the scaffold was in the form of a paving stone honeycomb, which was suitable for cell adhesion and growth, as depicted in Figure 1A–D. After co‐culturing for 5 days, the fibroblasts had essentially covered the scaffold, and the Calcein‐AM/PI staining saw that the cells were growing well, and only a small number of cells were dead (red fluorescence), most of them were surviving (green fluorescence), as shown in Figure 1E,F. These findings indicate that we have successfully prepared a well‐structured Astragalus polysaccharide‐containing 3D‐printed scaffold, which is suitable for cell growth and has no obvious toxicity to cells.

### 3.2 Results of laser Doppler perfusion and postoperative wound area

**FIGURE 2:**
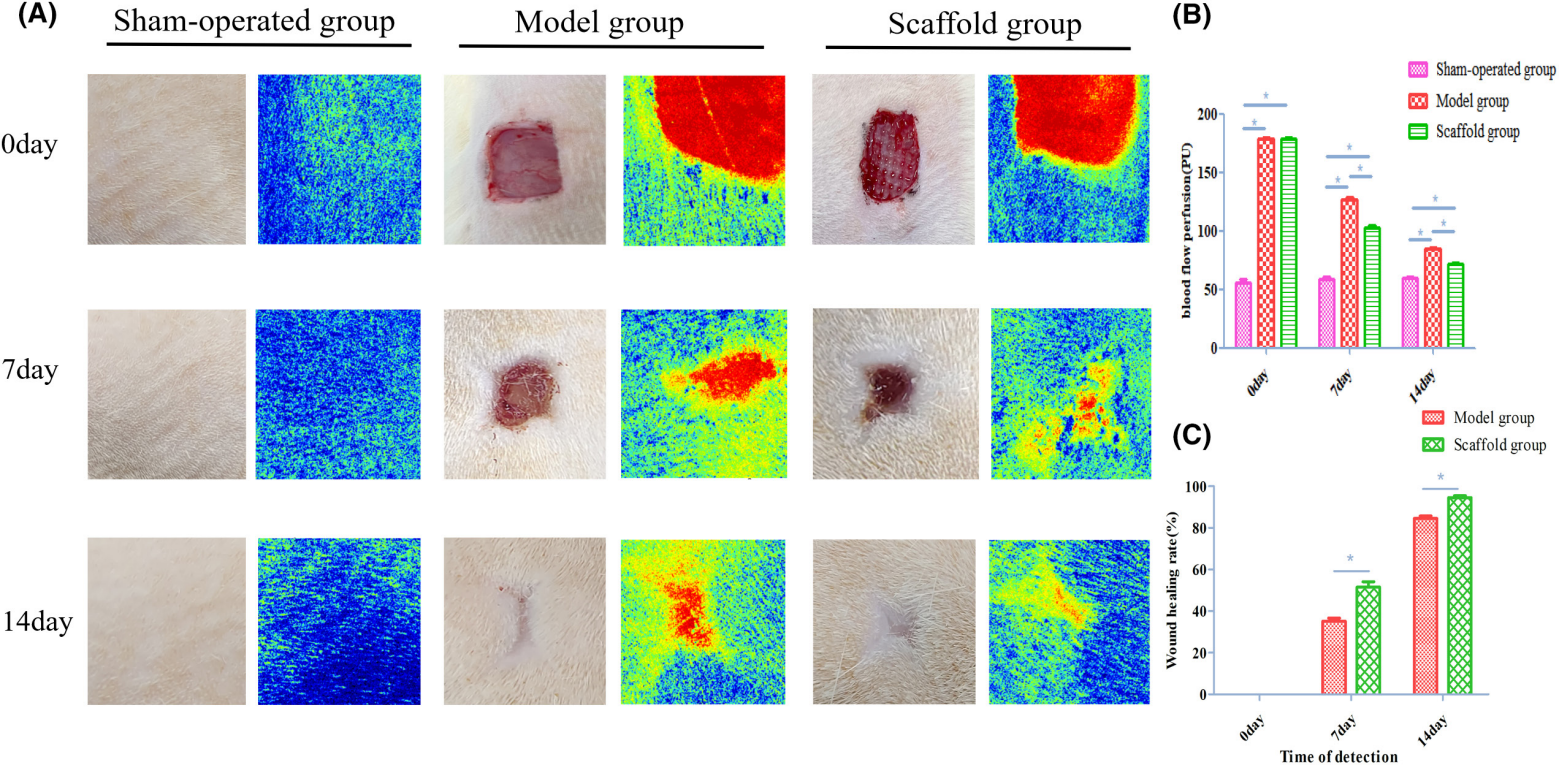
(A) Wound conditions and corresponding blood perfusion images of the three groups at different time points after surgery. (B) Comparison of wound blood perfusion volume in the three groups at different time points after surgery. (C) Comparison of wound healing rate between Model group and Scaffold group at different time points after surgery.

On the postoperative day, the blood perfusion in the model group and the scaffold group was comparable, but significantly higher than that in the sham‐operated group and the difference was statistically significant (*p* < 0.05). On the 7th postoperative day, the blood perfusion of the model group and the scaffold group gradually decreased, but the perfusion of the model group was still larger than that of the other two groups, and the difference was statistically significant (*p* < 0.05). On postoperative day 14, the blood perfusion of the scaffold group was closer to that of the sham‐operated group, and the difference was statistically significant (*p* < 0.05), and the results are shown in Figure 2A,B. The wound healing rate of the scaffold group was faster than that of the model group on postoperative days 7 and 14 and the difference was statistically significant (*p* < 0.05) and the results are shown in Figure 2C. The above illustrates that the Astragalus polysaccharide‐containing 3D‐printed scaffold improves traumatic blood perfusion and promotes trauma healing.

### 3.3 Results of histological and immunofluorescence detection

**FIGURE 3:**
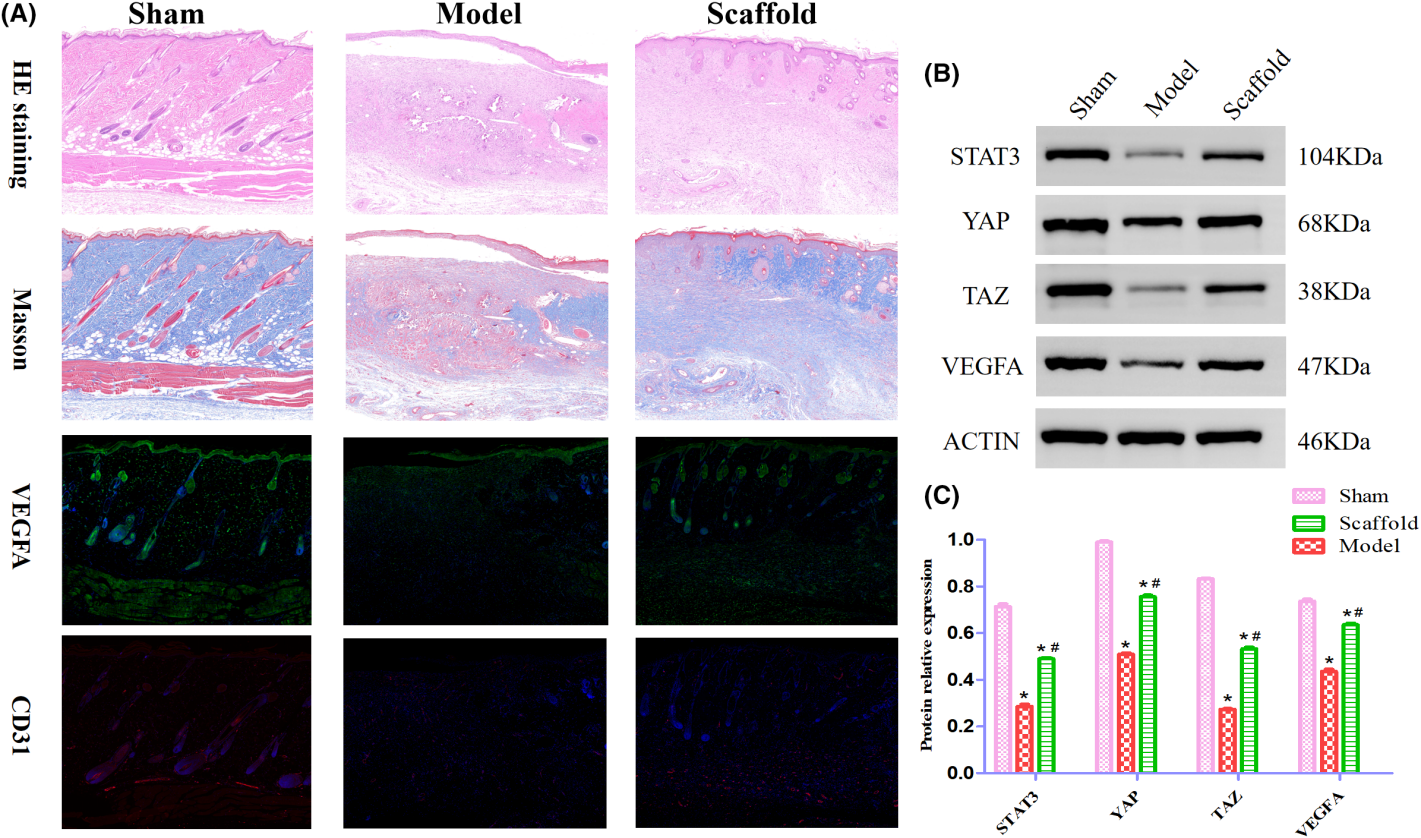
(A) Histological and immunohistochemical results of each group (X200). (B) Western blot was used to detect related proteins in each group and their grey values were compared.

On the 7th day after the operation, the epithelial cells and fibroblasts in the scaffold group were proliferated, collagen fibres were densely arranged, and a thinner neo‐epidermal layer was visible with some neo‐new hair follicle structures, while the model group had poorer skin repair than the scaffold group. The fluorescence expression of VEGFA and CD31 was better in the scaffold group than in the model group and the scaffold group was closer to the sham‐operated group. The results are shown in Figure 3A. The above indicates that Astragalus polysaccharide‐containing 3D printed scaffold can promote the structural repair of trauma, which may be related to the acceleration of traumatic neovascularisation.

### 3.4 Results of Western blot‐related protein detection

On the 7th day after the operation, the protein expression of STAT3, YAP, TAZ and VEGFA was highest in the sham‐operated group and gradually decreased in the scaffold group and model group (*p* < 0.05) and the protein expression of the scaffold group was better than that of the model group (*p* < 0.05), the results are shown in Figure 3B,C. The above suggests that an Astragalus polysaccharide‐containing 3D‐printed scaffold may with the aid of the nuclear entry of STAT3, activate the YAP/TAZ signalling pathway, promoting the VEGFA, accelerating the neovascularisation of the wound and promoting wound healing.

### 3.5 Results of differential protein analysis

#### 3.5.1 Volcano plot results for comparison of groups

**FIGURE 4:**
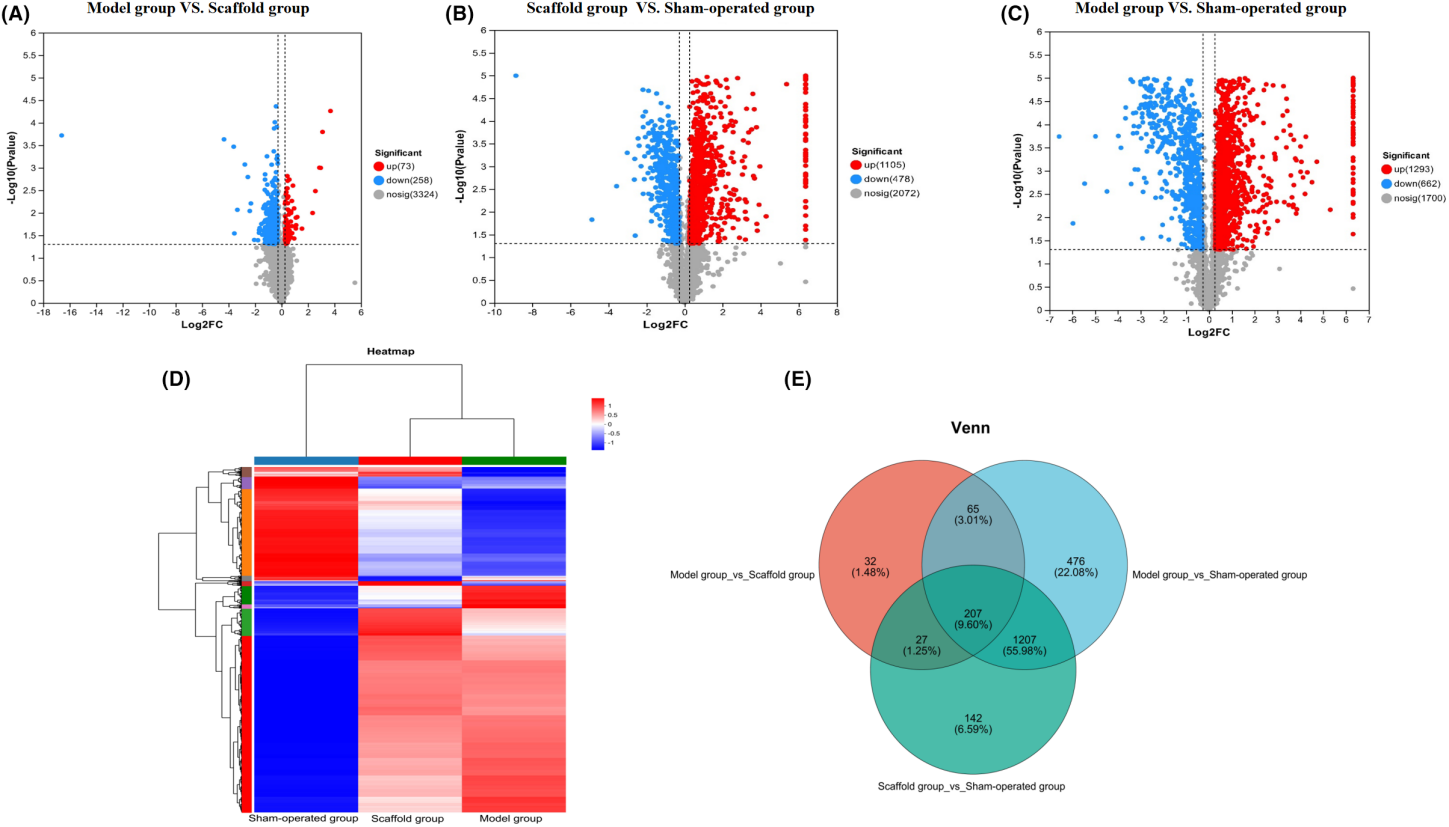
(A–C) Volcano diagrams showed results. The heat diagram showed the result. Venn diagram showed result.

The volcano plot in red indicates up‐regulated variables, blue indicates down‐regulated variables, and grey indicates variables with no difference. Model group versus scaffold group shows that 73 proteins were up‐regulated, and 258 proteins were down‐regulated (Figure 4A). The top 20 up‐regulated and down‐regulated proteins are listed in Table 1. The scaffold group versus sham‐operated group showed 1105 proteins were up‐regulated, and 478 proteins were down‐regulated (Figure 4B). The top 20 up‐regulated and down‐regulated proteins are listed in Table 2. Model group versus sham‐operated group showed that 1293 proteins were up‐regulated, and 662 proteins were down‐regulated (Figure 4C). The top 20 up‐regulated and down‐regulated proteins are listed in Table 3. The above suggests that Astragalus polysaccharide‐containing 3D‐printed scaffold may promote wound healing by reversing a certain degree of protein disorder.

**TABLE 1:** The top 20 proteins changed in the model group compared with the scaffold group.

Up‐regulation	Down‐regulation
Gpc6	Ndufv1
Adnp	Glipr2
Ptprf	Gcdh
Cald1	Slc25a12
Sp3	Ankrd2
Csrp1	Nt5c1a
Thbs2	Atp1b1
Npm1	Uqcrfs1
Lima1	Smtnl1
Nfxl1	Gpd1l
Twf1	Sdhb
Flna	Gpd2
Ptk7	Sbds
Cpxm1	Ndufb5
Fbln2	Tbxas1
Pls3	Mpi
Fbln1	Gamt
Marcksl1	Cacna1s
Tnc	Bin1
Ctsc	Prxl2b

**TABLE 2:** The top 20 proteins changed in the scaffold group compared with the sham‐operated group.

Up‐regulation	Down‐regulation
Mrpl37	Cpa3
Sash3	Atp1a2
Spp1	Prnp
Kcnab2	Col5a1
Plpp1	Ldb3
Akr1b8	Rpgr
Ptpro	Ppp1r3a
Klni	Ca3
Nckap1l	Dcn
Tcirg1	Actn3
Nol11	Ndufs4
Acaa1a	Ndufs7
Parp9	Mpst
Pgam1	Myl9
Vps4b	Eno3
Parp14	Acyp2
Coro1b	Myoz2
Hip1	Ola1
Lsm1	Samd9l
Adpgk	Syne1

**TABLE 3:** The top 20 proteins changed in the model group compared with the sham‐operated group.

Up‐regulation	Down‐regulation
Nckap1l	Syne1
Tsc1	Snta1
Rras2	Mylk2
Bst1	Actn3
Adnp	Atp1a2
Akr1b8	Ca3
Cib1	Rpgr
Cstf3	Macrod1
Pitpnb	Idh3g
Dock2	Mybpc2
Tardbp	Ldb3
Spon1	Hspb1
Me2	Sh3bgr
Pls3	Ewsr1
Cndp2	Neb
Zbtb8os	Trim72
Pfn1	Thbs2
Rab31	Pfkm
Gpc6	Klni
Car4	Pfkm

#### 3.5.2 Results of comparative hierarchical cluster analysis (HCA) plot for each group

The colour contrast between blue and red in the HCA diagram becomes increasingly distinct. The expression levels of numerous proteins in the model group are in contrast to those in the sham‐operated group. In the scaffold group, some proteins were reversed compared with the model group, and the protein expression level was closer to that of the sham‐operated group. See Figure 4D. The above further supports the results of the volcano plot.

#### 3.5.3 Results of Venn diagram comparisons between groups

The Venn diagram showed that there were 207 differential proteins in the three groups, 32 differential proteins specific to model group versus scaffold group, 476differential proteins specific to model group versus sham‐operated group, 142differential proteins specific to scaffold group versus sham‐operated group, 27differential proteins specific to model group versus scaffold group and scaffold group versus sham‐operated group. Group‐specific differential proteins 142, model group versus scaffold group shared 27 differential proteins with scaffold group versus sham‐operated group, model group versus sham‐operated group had a total of 65 differential proteins and scaffold group versus sham‐operated group had a total of 1270 differential proteins versus model group versus sham‐operated group. See Figure 4E.

### 3.6 Results of differential protein bioinformatics analysis

#### 3.6.1 Results of GO classification analysis histogram and enrichment analysis chronograms

**FIGURE 5:**
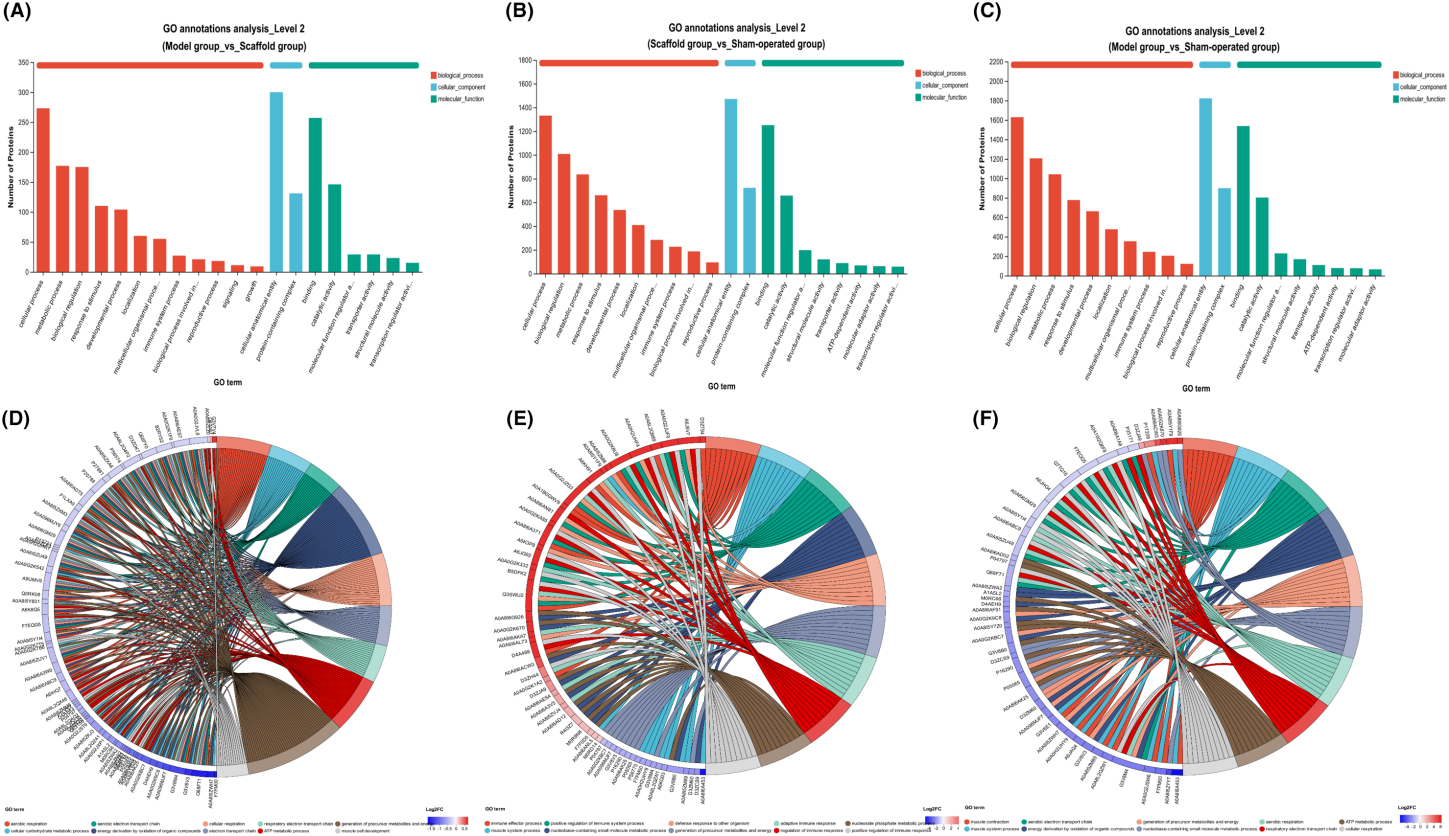
(A–C) Bar chart results of GO function classification. (D–F) Chordgram results of GO enrichment.

The first 20 entries with the smallest *p*‐value were taken for the GO classification analysis bar chart. The colours indicate the different categories: biological process, molecular function and cellular component, the vertical coordinate indicates the number of proteins in each category and the horizontal coordinate indicates the specific protein groups. The GO classification analysis bar chart shows (Figure 5A–C) that: cellular process, biological regulation, metabolic process, cellular anatomical entity, protein‐containing complex, binding and catalytic activity are the groups of differential protein changes in the major classifications. Enrichment analysis of chordal plots showed (Figure 5D–F): that mitochondrial function, immune response, redox response, extracellular gap and ATP metabolic process were the major groups of differential protein changes.

#### 3.6.2 Results of the bar chart for KEGG classification analysis and the bubble chart for enrichment analysis

**FIGURE 6:**
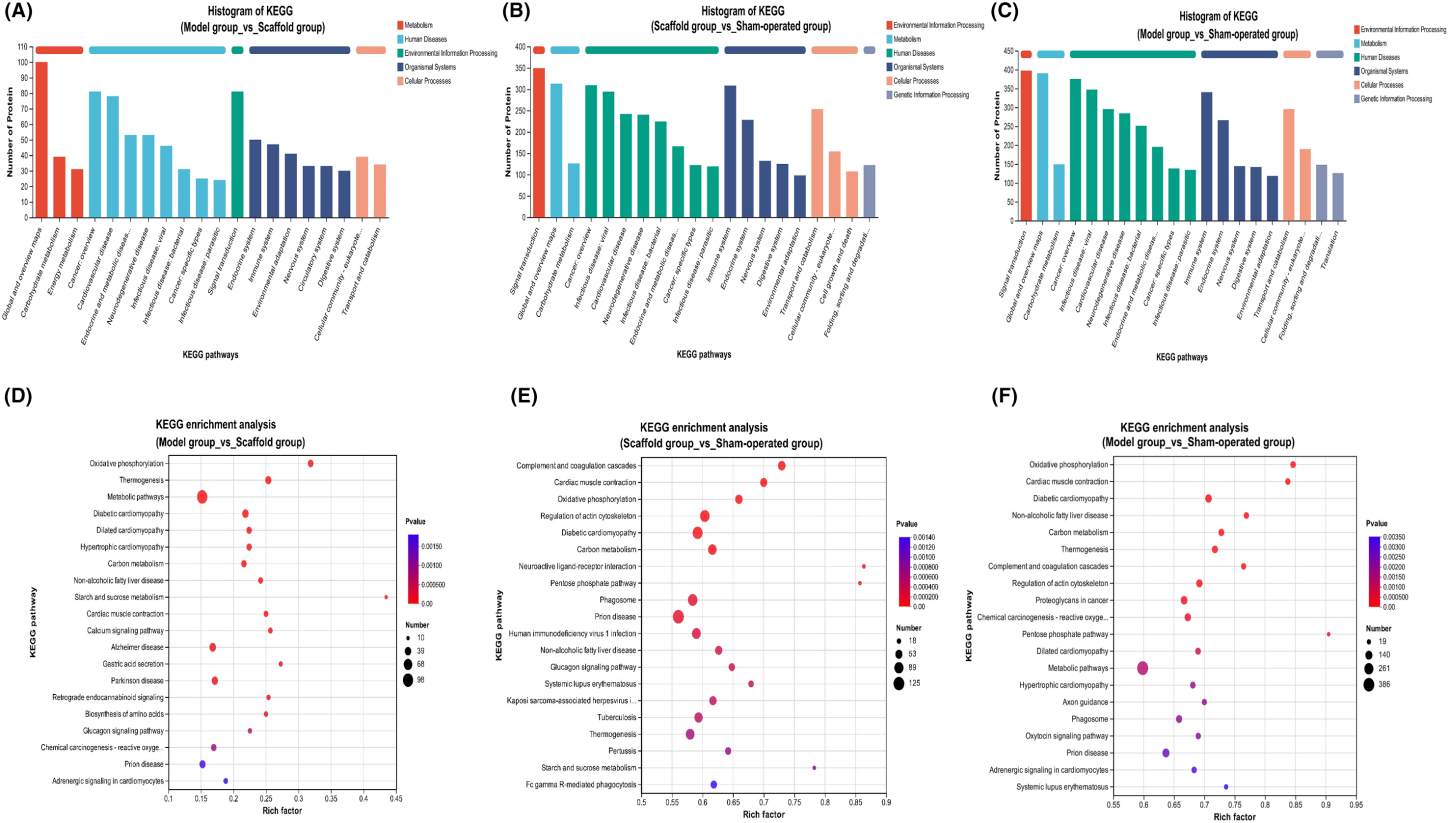
(A–C) Bar chart results of KEGG pathway classification. (D–F) Bubble diagram results of GO enrichment.

The first 20 entries with the smallest *p*‐value were taken for the KEGG classification analysis bar chart. The colours indicate different classifications: metabolism, genetic information processing, environmental information processing, cellular processes, organismal systems, human diseases and drug development. The *y*‐axis indicates the number of proteins in each classification and the *x*‐axis indicates specific pathway groups. The KEGG classification analysis bar graphs show (Figure 6A–C) that signal transduction, energy metabolism, carbohydrate metabolism and the immune system are the groups of differentiated pathway variations in the major classifications. Enrichment analysis bubble plots show (Figure 6D–F): that oxidative phosphorylation, metabolic pathways, carbon metabolism, calcium signalling pathways, etc. are the major groups of differential metabolic pathway changes.

## 4 DISCUSSION

The physiological functions of various parts of the skin are inextricably linked to the physiological structure and function of the basic skin compounds, including proteins, lipids, carbohydrates and nucleic acids. These molecules have a wide variety of functions, such as enzyme activity and signalling.^25^ ECM scaffolds have been demonstrated to promote cell differentiation, proliferation, vascular neovascularisation and wound healing.^26^, ^27^ Proteins are key substances in the proliferation, migration and differentiation of epidermal cells, fibroblasts and immune cells. They are essential for angiogenesis and collagen synthesis and are involved in the whole process of wound healing, which is a key factor affecting its healing.^28^, ^29^ In this study, we demonstrated that an Astragalus polysaccharide‐containing 3D‐printed scaffold could accelerate skin wound repair by activating the YAP/TAZ signalling pathway, promoting vascular regeneration and correcting protein disorders.

Bio‐3D printing technology has shown a broad application prospect in the field of skin repair materials and is highly reproducible.^30^ Astragalus polysaccharide is the main active substance of Astragalus, which has good anti‐inflammatory, vasculoprotective and angiogenesis‐promoting effects. Zhang et al. found through experimental studies that Astragalus could promote functional recovery by accelerating angiogenesis through activation of the AKT/eNOS signalling pathway.^23^ Another study found that Astragalus polysaccharide could inhibit the expression of IκBα and cyclin D1 in HSF cells, promote the proliferation, migration and cycle progression of HSF cells, and promote the re‐epithelialization, vascular reconstruction and cytokine secretion of TGF‐β1, bFGF and EGF.^31^ Our prepared Astragalus polysaccharide‐containing 3D‐printed scaffold was shown under scanning electron microscopy: the scaffold was a white transparent lattice‐like structure with consiscaffold pore size, spacing and thickness, presenting a paving‐stone‐like honeycomb surface, a structural feature that facilitates cell adhesion and growth. After co‐culturing the scaffold with fibroblasts for 5 days, we observed that the cells had covered the whole scaffold and most of them remained active. It demonstrated that our prepared Astragalus polysaccharide‐containing 3D‐printed scaffold not only promotes cell growth but also has no obvious toxic effects on cells.

Angiogenesis is a key link in wound healing, and the rate of local vascular regeneration determines the effectiveness of tissue repair.^32^, ^33^ VEGF can effectively promote the growth of neovascular endothelial cells, and it is the most widely studied angiogenic factor in the process of wound healing.^34^ Sun et al. maintained the local concentration of VEGF in the wound by a polymer slow‐release system in a mammalian hindlimb ischemia model, thus observing more obvious angiogenesis.^35^ In our study, we found that VEGFA protein expression was higher in the scaffold group than in the model group, and the recovery of blood perfusion in the scaffold group was found to be superior to that in the model group as detected by Doppler perfusionmetry. The results suggest that an Astragalus polysaccharide‐containing 3D printed scaffold can improve local tissue blood flow micro‐circulation in rats with total skin defects, and can accelerate wound neovascularisation and promote wound healing by promoting VEGFA expression.

Recent studies have shown that the Hippo signalling pathway has an important regulatory role in cell proliferation and differentiation.^36^, ^37^ YAP and TAZ are downstream effectors of the Hippo signalling pathway.^38^, ^39^ During skin repair, YAP/TAZ can effectively promote the proliferation and migration of basal lamina cells and fibroblasts, and accelerate the repair of defective tissues. Some studies have found that fibroblasts can transmit changes in peripheral mechanical signals with the help of YAP/TAZ, which drives the activation of fibroblasts and promotes the secretion of collagen fibres.^40^ Guo et al. found that platelet‐rich plasma‐derived exosomes promote the proliferation and migration of fibroblasts through the activation of YAP/TAZ, which in turn promotes the healing of difficult‐to‐heal wounds.^41^ YAP/TAZ the downstream of the Hippo signalling pathway is an extremely critical regulatory factor in vascular development. During development, YAP/TAZ in blood vessels is activated, thus promoting angiogenesis. YAP/TAZ are important transcription factors that need to enter the nucleus to function. Some researchers have found that YAP/TAZ, which binds to STAT3, can enter the nucleus with the help of STAT3's nuclear localization signal, thus activating the transcriptional program of angiogenesis.^42^ It has also been shown that activated YAP interacts with STAT3, blocking VEGF expression and promoting endothelial barrier repair.^43^ In contrast, the angiogenic process was significantly prolonged in adult mice after the knockdown of YAP/TAZ.^44^ In our study, we found that the expression of STAT3, YAP and TAZ proteins in the scaffold group was higher than that in the model group, suggesting that the Astragalus polysaccharide‐containing 3D‐printed scaffold may have activated the YAP/TAZ signalling pathway with the help of STAT3 into the nucleus, promoting fibroblast differentiation, further restoring the skin tissue homeostasis and accelerating the wound healing.

Proteomics, as a key research tool, complements and refines our understanding of genomic data. Proteomics technology allows us to qualitatively and quantitatively analyse the proteins involved in the wound healing process, thus revealing the key proteins and their interactions related to healing.^45^ Hsiao et al. treated fibroblasts with comfreyin and identified 22 differentially expressed proteins, whose functions are related to antioxidant activity, anti‐apoptotic activity, modulation of cellular motility and secretion of collagen, promotion of cell proliferation, among others.^46^ However, in our study, we found that the groups of differentially expressed proteins changes in mitochondrial function, immune response, redox response, extracellular gap and ATP metabolic processes were significantly dialled back after Astragalus polysaccharide‐containing 3D‐printed scaffold treatment. Among them, the callback of mitochondrial function will effectively increase ATP synthesis, which may effectively inhibit excessive inflammation and redox response, and also promote angiogenesis. Oxidative phosphorylation, metabolic pathways, carbon metabolism, calcium signalling and other pathways related to differential metabolic pathway change groups were significantly regulated. Among them, the callback of the oxidative phosphorylation pathway can reverse the hypoxia state of mitochondria and improve ATP production, which has a protective effect on cells. The calcium signalling pathway is important for keratinocyte differentiation, improvement of skin permeability and reconstruction of skin barrier. It suggests that an Astragalus polysaccharide‐containing 3D‐printed scaffold may promote skin wound healing by reversing protein disorders. Our proteomic analysis provides new ideas to reveal the regulatory function of Astragalus polysaccharide‐containing 3D‐printed scaffold in wound healing.

**FIGURE 7:**
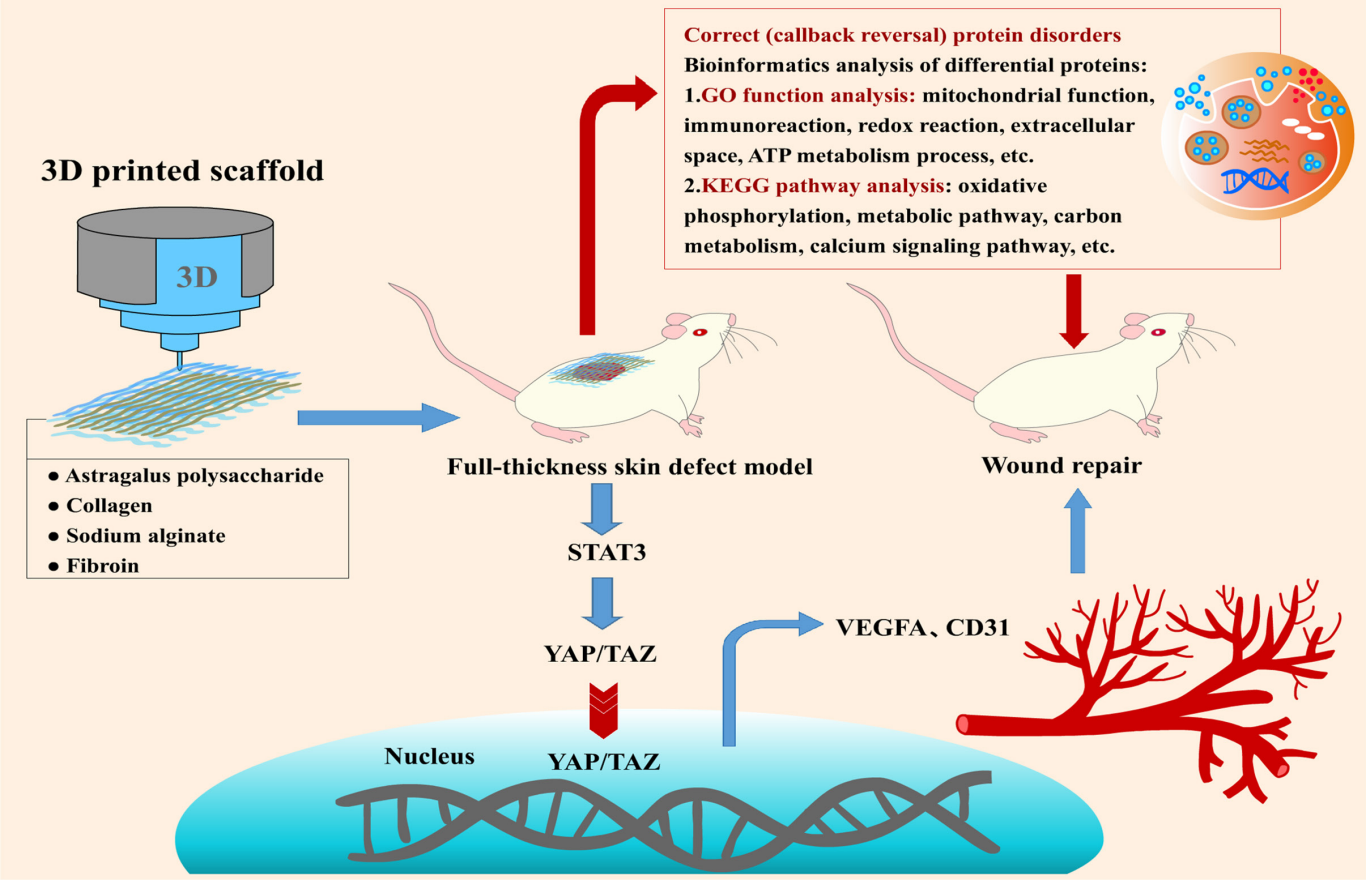
Schematic diagram of the repair effect of 3D printed scaffold containing Astragalus polysaccharide on wound skin and the research results of proteomics.

In summary, Astragalus polysaccharide‐containing 3D‐printed scaffold can reverse protein disorder and may play a key role in the mechanism of action of Astragalus polysaccharide‐containing 3D‐printed scaffold to promote skin wound repair. Astragalus polysaccharide‐containing 3D‐printed scaffold may activate the YAP/TAZ signalling pathway with the help of STAT3 nucleation, promote the restoration of blood perfusion and vascular regeneration during wound repair in rats, and promote the proliferation of epithelial cells and fibroblasts in rats, to achieve wound repair (see Figure 7). Although there are some limitations, such as the mechanism of action of Astragalus polysaccharide‐containing 3D‐printed scaffold on skin proteomics and YAP/TAZ signalling pathway needs to be verified by more experimental studies, and there have not yet been any clinical trials to validate their efficacy. However, our findings may provide new ideas for the treatment of skin wound repair and provide an experimental basis for subsequent clinical trials.

## AUTHOR CONTRIBUTIONS

**Weibin Du:** Conceptualization (lead); data curation (lead); project administration (lead); resources (lead); writing – original draft (lead). **Zhenwei Wang:** Conceptualization (equal); data curation (equal); writing – original draft (equal). **Meichun Han:** Conceptualization (equal); data curation (equal); writing – original draft (equal). **Yang Zheng:** Formal analysis (supporting); methodology (supporting); validation (supporting). **Bowen Tao:** Data curation (supporting); formal analysis (supporting); validation (supporting). **Ningfang Pan:** Data curation (supporting); formal analysis (supporting); validation (supporting). **Guanai Bao:** Data curation (supporting); formal analysis (supporting); funding acquisition (supporting). **Wei Zhuang:** Conceptualization (supporting); supervision (supporting); writing – review and editing (supporting). **Renfu Quan:** Methodology (lead); project administration (lead); supervision (lead); writing – review and editing (lead).

## FUNDING INFORMATION

This research was supported by the National Natural Science Foundation of China (no. 81904053). Zhejiang Provincial Natural Science Foundation of China under Grant (no. LTGY24H290006). Special Research Project of the Affiliated Hospital of Zhejiang Chinese Medical University (no. 2021FSYYZY43). The Medical and the Health Science Project of Zhejiang Province (no. 2021KY577). Hangzhou Medical and Health Technology Planning Project (no. B20220021), Hangzhou Science and Technology Planning Project (no. 20220919Y084). Zhejiang Province Traditional Chinese Medicine Science and Technology Project (no. 2023ZR046). Major science and technology project of Xiaoshan District (no. 2019216).

## CONFLICT OF INTEREST STATEMENT

The authors declare no conflicts of interest.

## Data Availability

All data generated and/or analysed during this study are included in this published article.
